# Mechanically Stable Magnetic Metallic Materials for Biomedical Applications

**DOI:** 10.3390/ma15228009

**Published:** 2022-11-12

**Authors:** Shahid Mehmood, Zahid Ali, Shah Rukh Khan, Salma Aman, Ashraf Y. Elnaggar, Mohamed M. Ibrahim, Tatiana I. Zubar, Daria I. Tishkevich, Sergei V. Trukhanov, Alex V. Trukhanov

**Affiliations:** 1 Department of Physics, Center for Computational Materials Science, University of Malakand, Chakdara, Dir (Lower) 18800, Pakistan; 2 Institute of Physics, KhwajaFareed University of Engineering and Information Technology, Abu Dhabi Road, Rahim Yar Khan 64200, Pakistan; 3 Department of Food Science and Nutrition, College of Science, Taif University, P.O. Box 11099, Taif 21944, Saudi Arabia; 4 Department of Chemistry, College of Science, Taif University, P.O. Box 11099, Taif 21944, Saudi Arabia; 5 Laboratory of Magnetic Films Physics, Scientific-Practical Materials Research Centre of National Academy of Sciences of Belarus, 220072 Minsk, Belarus; 6 Laboratory of Single Crystal Growth, South Ural State University, 454080 Chelyabinsk, Russia; 7 Smart Sensors Laboratory, Department of Electronic Materials Technology, National University of Science and Technology MISiS, 119049 Moscow, Russia

**Keywords:** anti-perovskites, biomaterials, electronic properties, elastic properties, magnetic properties

## Abstract

The structural, electrical, and magneto-elastic properties of lanthanide base nitride (Ln = Dy-Lu) anti-perovskites were investigated using density functional theory (DFT). The reported structural outcomes are consistent with the experiment and decrease from Dy to Lu due to the decrease ofatomic radii of Ln atoms. According to the electronic band profile, the metallic characteristics of these compounds are due to the crossing over of Ln-f states at the Fermi level and are also supported by electrical resistivity. The resistivity of these compounds at room temperature demonstrates that they are good conductors. Their mechanical stability, anisotropic, load-bearing, and malleable nature are demonstrated by their elastic properties. Due to their metallic and load-bearing nature, in addition to their ductility, these materials are suitable as active biomaterials, especially when significant acting loads are anticipated, such as those experienced by such heavily loaded implants as hip and knee endo-prostheses, plates, screws, nails, dental implants, etc. In thesecases, appropriate bending fatigue strength is required in structural materials for skeletal reconstruction. Magnetic properties show that all compounds are G-type anti-ferromagnetic, with the Neel temperatures ranging from 24 to 48 K, except Lu_3_Nin, which is non-magnetic. Due to their anti-ferromagnetic structure, magnetic probes cannot read data contained in anti-ferromagnetic moments, therefore, data will be unchanged by disrupted magnetic field. As a result, these compounds can be the best candidates for magnetic cloaking devices.

## 1. Introduction

Different combinations of elements produced rich metallic perovskites and anti-perovskites. Like the perovskite crystal structure frequently seen in nature, anti-perovskites are also a form of this crystal structure [1]. The primary distinction is that the cation and anion constituent positions are flipped in the structure of the unit cell. Anti-perovskite compounds, in contrast to perovskites, are made up of two different anions coordinated with a single distinct cation. Because they possess intriguing and practical physical qualities not seen in perovskite materials, anti-perovskite compounds, which can be used as electrolytes in solid-state batteries, are a significant family of materials [2]. Anti-perovskiteshave the simple configuration of (M_3_Y)N (M = Transition Metal, and Y = C, N, O). Iron is composed of nitrides of numerous 3-d orbitals; in particular, the compound (Fe_3_Z)N has received attention due to its electrical characteristics [3,4,5,6] and its cubic phase anti-perovskites symmetry. However, they can be converted to orthorhombic perovskite structures, such as (X_3_N)Y (X = alkaline earth metals and Y = P-Block elements) [7,8,9]. The majority of compounds have semiconducting characteristics in the extensive variety of metal-rich perovskite. The electronic equilibrium in these perovskites is disrupted when the oxidation state of alkaline earth metals is replaced by +2 by +3, making them inherently metal, in addition to their larger magnetic moments [10,11,12]. Due to their numerous applications as sensors, detectors, electronic devices, optical devices, radiation, superconductivity, and energy devices, a substantial amount of research on rare-earth ions’ unique optical, magnetic, and electrical properties has recently been performed [13,14,15,16]. Therefore, rare-earth-containing compounds are selected to investigate the corresponding possibility to obtain information on electron-rich systems and to determine the possibility of introducing nuclei with localized magnetic moments.

There is not much information on the physical properties of ternary nitrides with the formula R_3_NIn (R = rare-earth metal). Only the structural properties of these compounds have been experimentally reported [11,17], while the physical properties of R_3_NIn (R = Ce, Pr, and Nd) have been theoretically reported [18]. Structure parameters are experimentally reported for the anti-perovskites Ln3NIn (Ln = Dy-Lu) compounds in this series, which exist in cubic symmetry with the Pm-3m space group and have lattice parameters ranging from 4.979 to 4.812 Å. The distance between the rare-earth elements, their electronic arrangement, and the oxidation state within the compounds in anti-perovskite nitrides influence their magnetic properties. Although these compounds have been experimentally synthesized [11,17], there is no information available regarding their electronic, elastic, magnetic, or electronic properties. As a result, the current study aims to examine the structural, elastic, magnetic, and electronic properties of the understudy compounds using density functional theory because of their ability to withstand tensile stresses, exhibit metallic and ductile nature, and be used as biomaterials for skeletal reconstructions spatially when large acting loads are predictable.

## 2. Materials and Methods

**Figure 1:**
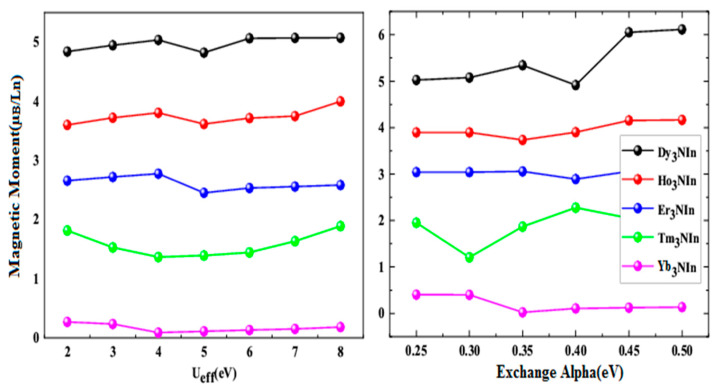
(**a**) optimization of Hubbard U, (**b**) exchange alpha against magnetic moment of Ln atom of rare earth nitrides Ln_3_NIn (Ln = Dy-Lu) anti-Perovskite.

The structural, electronic, magnetic, and elastic properties of the Ln_3_NIn (Ln = Dy-Lu) lanthanide anti-perovskites nitrides were investigated utilizing the full-potential linear augmented plane wave method in the scaffold of DFT [19,20] using the WIEN2k software. In order to treat the correlation of the Ln-4f state electrons, the PBEsol functional contained by the Generalized Gradient Approximation (GGA) [21], GGA with Hubbard U (GGA+U) [22], and the B3PW91 flavor of hybrid function (HF) [23] were used. The Hubbard U value and exact exchange α were optimized in terms of magnetic moment per Dy-Lu atom, as shown in Figure 1a,b.

The optimal values of U for Ln are 5 eV/Dy, but for Ho and Er, are 4eV/Tm and Yb, and the exchange is 0.30 eV for Tm, 0.35 for Ho and Yb, and 0.40 for Dy and Er. The following values have been used in the overall calculation for the proper treatment of the Ln-f state to take apart these compounds’ valence and core states, and the partition energy was taken as 7.0 Ry.The GGA+U approach was ultimately chosen among the many methods or approximations because it fitted well to measure the electronic exchange with correlation potentials. Different factors were taken by default in these calculations to obtain consistent findings, such as L_max_ = 10 Ry, RK_max_ = 7 Ry, and G_max_ = 12 Ry. K_max_ × R_MT_ = 8 is utilized to provide exact and well-converged findings. In between each iteration, the energy is reduced to 0.1mRy, the forces are reduced to 1mRy Bohr-1, and 10 × 10 × 10 K-points are employed in the Brillouin Zone. The BoltzTrap code [24], based on semi-classical Boltzmann theory incorporated into WIEN2k software, was used to compute the thermoelectric parameters (resistivity and magnetic susceptibility) of compounds. At the same time, the IRelast package [25] was utilized to determine the elastic characteristics.

## 3. Results and Discussion

### 3.1. Structural Properties

**Figure 2:**
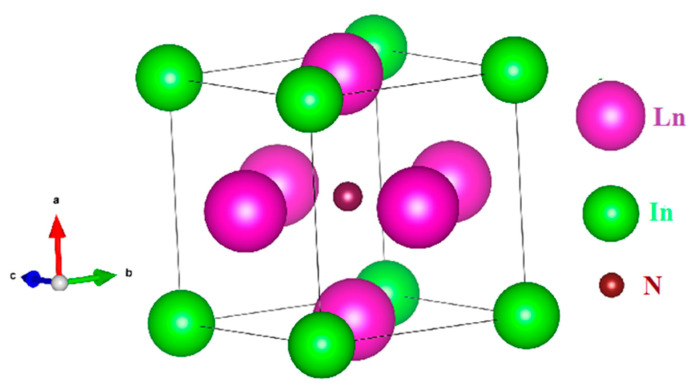
Unite cell structure of the rare earth nitrides Ln_3_NIn (Ln = Dy-Lu) anti-Perovskite.

In the Pm-3m space group shown in Figure 2, rear earth Nitride anti-perovskites Ln_3_NIn (Ln = Dy-Lu) are produced in cubic phase.

**Figure 3:**
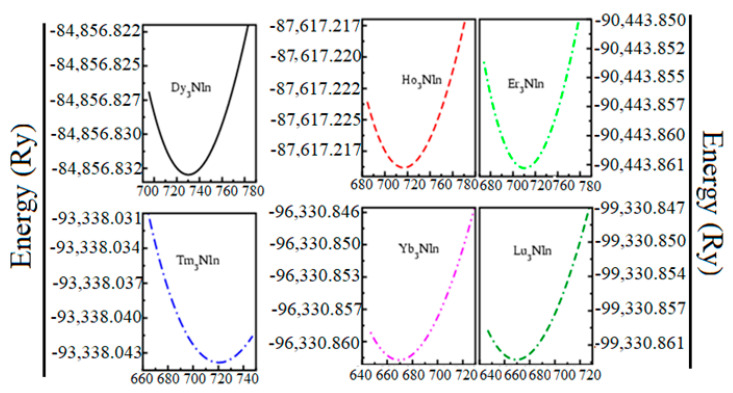
Energy versus volume optimization curve of the rare earth nitrides Ln_3_NIn (Ln = Dy-Lu) anti-Perovskite.

Experimental structure parameters [11] were used to optimize the structural geometry of these compounds in the scaffold of DFT employing GGA, GGA+U, and HF. To determine the ground state physical parameters, the ground state energy against the ground state volume of each unit cell is calculated by using the Birch–Murnaghan equation of state [26], as shown in Figure 3. The ground state structural parameters, such as ground state energy (E_o_), lattice constants (a_o_), cohesive energy (E_c_), and enthalpy of formation (ΔH), are presented in Table 1.

**Figure 4:**
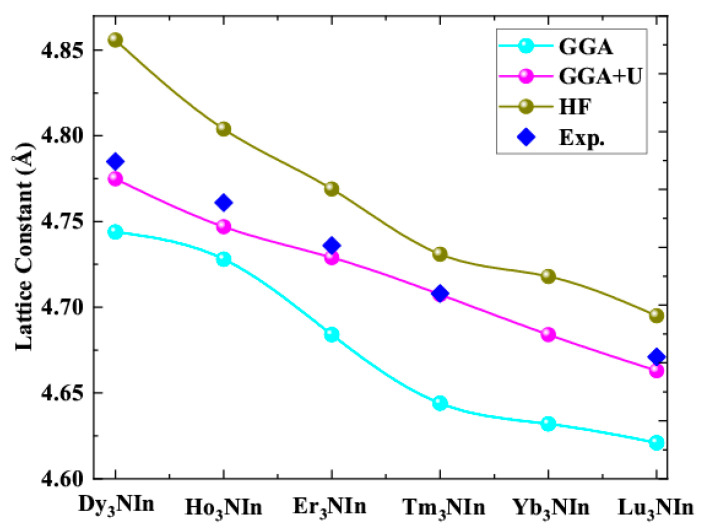
Comparison of calculated and experimental lattice constants of the rare earth nitrides Ln_3_NIn (Ln = Dy-Lu) anti-Perovskite.

From Table 1, the computed lattice constants for Dy_3_NIn are in the range of 4.744 to 4.856, for Ho_3_NIn are in the range of 4.728 to 4.804, for Er_3_NIn are in the range of 4.684 to 4.769, for Tm_3_NIn are in the range of 4.644 to 4.731, for Yb_3_NIn are in the range of 4.632 to 4.718, and for Lu_3_NIn are in the range of 4.4.621 to 4.695. The ground state energies for Dy_3_NIn are in the range of −84,856.83297 to −84,856.53872 Ry, for Ho_3_NIn are in the range of −87,617.534213 to −87,617.12521 Ry, for Er_3_NIn are in the range of −90,444.208576 to −90,443.208576 Ry, for Tm_3_NIn are in the range of −93,338.043837 to −93,337.59376Ry, for Yb_3_NIn are in the range of −96,301.085485 to −96,299.93979Ry, and for Lu_3_NIn are in the range of −99,330.92911 to −99,330.78657Ry. The comparison between the estimated and experimental lattice constants is plotted in Figure 4 and given in Table 1, which demonstrates that the given results are in accordance with the experiments [11,17].

From Figure 4, the lattice parameters obtained by GGA+U are closer to experiments and more reliable than the GGA and HF potentials.

The lattice constants are decreasing for the understudy compounds from Dy to Lu due to changes in atomic radii of Dy to Lu atoms in Ln_3_NIn (Ln = Dy-Lu), as seen from Figure 4.

E_coh_ and ΔH are critical stability parameters that have been derived [27] from E_0_ and are presented in Table 1. The higher E_coh_ of the system is due to greater Coulomb interactions between electrons and nuclei, indicating that it is strongly connected and well-integrated. Higher the E_coh_, the more tightly bound the system due to electron nuclei contact, which is not affected by the size or quantity of constituent particles in a compound. E_coh_ ranges from −32.11132 to −36.10071 for compounds Ln_3_NIn (Ln = Dy-Lu) and ΔH and from −6.422264 to −7.220142 for compounds Ln_3_NIn (Ln = Dy-Lu). Their thermodynamic stability was confirmed by the negative values of E_coh_ and ΔH in Table 1. From the E_coh_ and ΔH the compound Lu_3_NIn possesses greater value than the rest, indicating that this compound is more stable than all other compounds.

**Figure 5:**
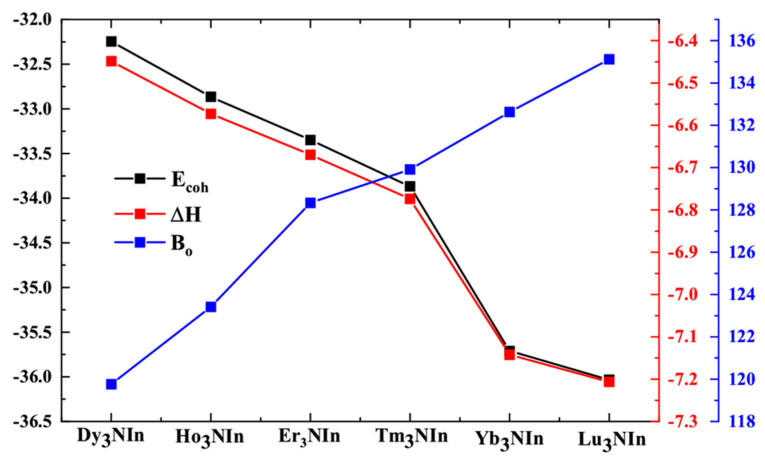
Cohesive energy, enthalpy of formation in comparison with the bulk modulus of the rare earth nitrides Ln_3_NIn (Ln = Dy-Lu) anti-Perovskite.

Cohesive energy and enthalpy of formation are plotted in comparison with bulk modulus for compounds Ln_3_NIn (Ln = Dy-Lu) [28] from Table 1 and Table 2 and shown in Figure 5.

In Figure 5, all the stability parameters are increased going from Dy to Lu base compounds. It shows that the hardness of these compounds is increased from Dy-Lu and discloses the more stable nature of Lu_3_NIn.

### 3.2. Electronic Properties

**Figure 6:**
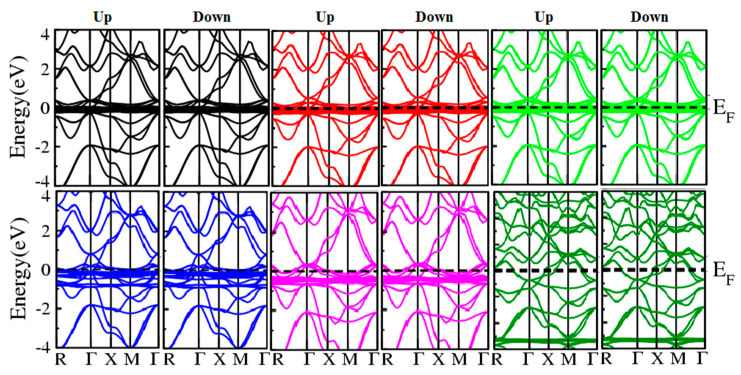
Electronic band structure of the rare earth nitrides Ln_3_NIn (Ln = Dy-Lu) anti-Perovskite. Black, red, green, blue, magneta and dark green colors indicate the band structure for Dy_3_NIn, Ho_3_NIn, Er_3_NIn, Tm_3_NIn, Yb_3_NIn and Lu_3_NIn.

The electronic characteristics of these Ln_3_NIn (Ln = Dy-Lu) are estimated by HF potential using electronic band structures for these compounds in the anti-ferromagnetic configuration presented in Figure 6.

**Figure 7:**
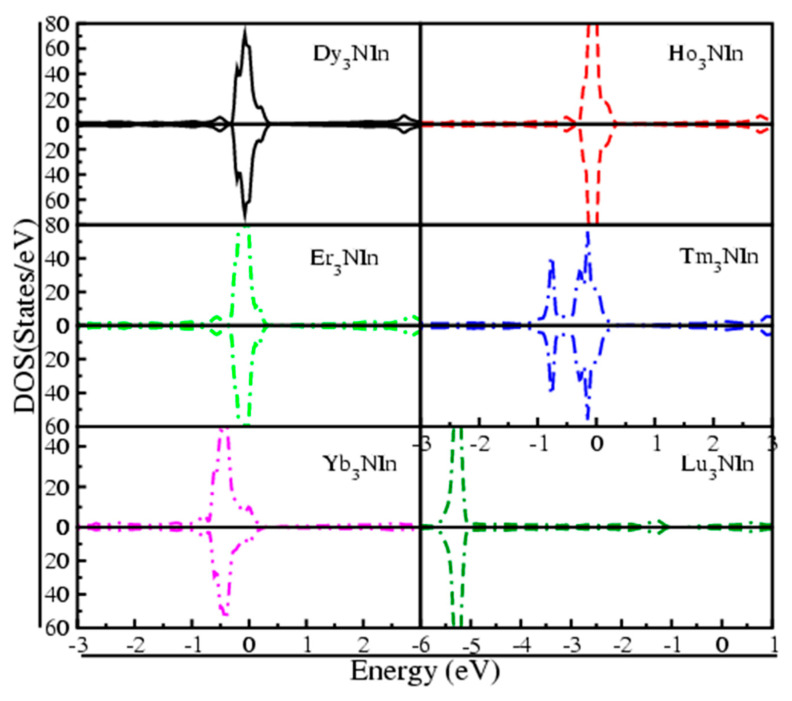
Total DOS of the rare earth nitrides Ln_3_NIn (Ln = Dy-Lu) anti-Perovskite.

From Figure 6, it is evident from the valence band (VB) and conduction band (CB), which demonstrate their metallic nature, such that at the Fermi level (E_F_), the states of all compounds are overlapping. The reported metallic nature of the understudy compounds is in accordance with experimental results [11,17]. To verify the results estimated from the band structure of these compounds, their total density of states (TDOS) is also considered to study the basis of band structure. Figure 7 shows the calculated TDOS for these compounds and demonstrates that the densities are lengthened from the VB to CB, crossing the Fermi level, indicating that the compounds under investigation are metallic.

**Figure 8:**
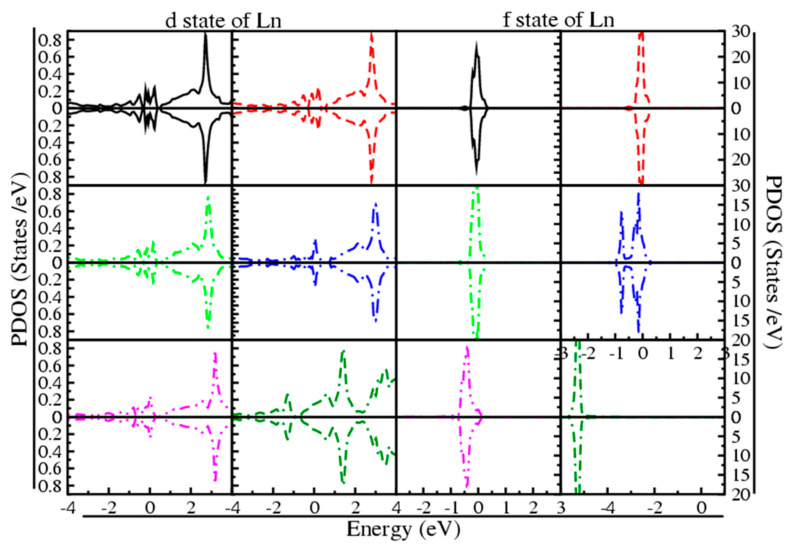
Partial DOS of the rare earth nitrides Ln_3_NIn (Ln = Dy-Lu) anti-Perovskite. Black, red, green, blue, magneta and dark green colors indicate the d and f state for Dy_3_NIn, Ho_3_NIn, Er_3_NIn, Tm_3_NIn, Yb_3_NIn and Lu_3_NIn.

The contribution of Ln-d and f states to their metallic nature is also observed from their PDOS, as shown in Figure 8.

From the graph, it is obvious how the Ln-d and f states are involved in the metallic nature of all of these compounds. Except from Lu_3_Nin, where only the Lu-d state is responsible for their metallic character. Figure 8 demonstrates that the contribution of the d state is less in VB as compared to CB in both spin up and down states, and overlaps the E_F_, whereas the f state shows a contribution in both up and down states in VB and CB at the E_F_ (located at 0 eV), except from Lu_3_Nin, where the f state of Lu occurs at −5.3 eV in both VB and CB. It is concluded that the Ln-f state plays a greater role as compared to the Ln-d state for all these compounds in their metallic nature, except in Lu_3_NIn.

**Figure 9:**
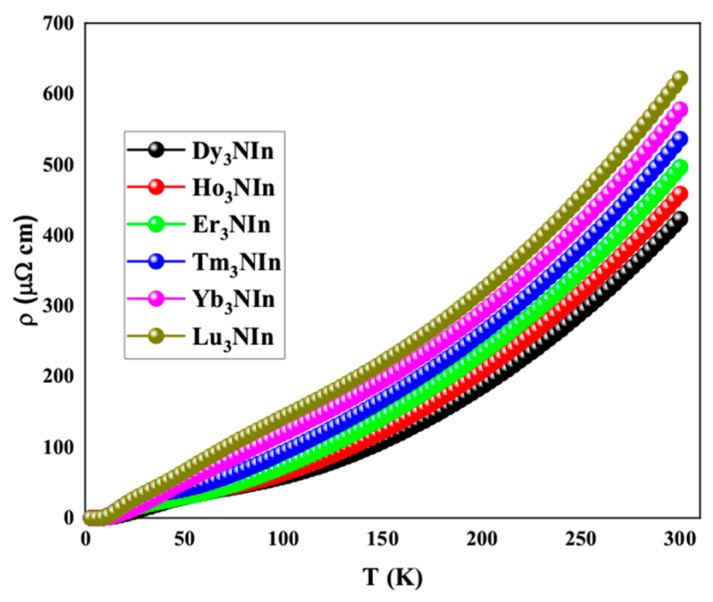
Electrical resistivity of the rare earth nitrides Ln_3_NIn (Ln = Dy-Lu) anti-Perovskite.

The electrical resistivity for Ln_3_NIn (Ln = Dy-Lu) is calculated using the BoltzTraP package [24] up to a 300 K temperature and presented in Figure 9.

It can be observed from the graph that the electrical resistance of all compounds increases as the temperature rises (Figure 9). As the temperature rises, the thermal velocity of electrons increases, resulting in more collisions between free electrons and an increase of resistance. At room temperature, the electrical resistivity of Nd_3_NIn is 241.90 cm, Pm_3_NIn is 268.75 cm, Sm_3_NIn is 296.82 cm, Eu_3_NIn is 326.10 cm, Gd_3_NIn is 356.69 cm, and Tb_3_Nin is 388.78 cm. The resistivity of Ln_3_NIn (Ln = Dy-Lu) increases from Dy to Lu as temperature rises, which is consistent with the isotropic compounds [11]. The increasing nature of electrical resistivity with a rise in temperature is a characteristic of metallic compounds. As a result, their electrical resistance supports the metallic character of these compounds as determined by their electronic properties.

### 3.3. Elastic Properties

The mechanical properties and elastic constants (Cij) of a structural solid demonstrate how it responds to external stress, and specific boundary factors are associated with a product’s functional applications. We clearly understand how the structural material responds to applied stress between the elastic ranges thanks to the stiffness coefficients Cij of stable structural materials, which are elastic constants. Furthermore, a good understanding of the elastic constants of structural materials is necessary for several practical applications involving the solid’s mechanical properties, such as thermo-elastic stress, load deflection, internal strain, fracture toughness, and sound velocities. Solids’ fundamental elastic and mechanical properties are closely related to the types of anisotropic indices, mechanical stability, and crystal symmetry orientations [20].

IRelast, a package imbedded in WIEN2k software, calculates the elastic properties of the perovskites Ln_3_NIn (Ln = Dy-Lu) presented in Voigt notation Cij (GPa) = C_11_, C_12_, and C_44_ in Table 2. Mechanical parameters such as shear, Reuss shear, and bulk modulus (G_H_, G_R,_ B_o_), Paugh ductility index (B_o_/G_H_), Young’s modulus (Y), Poisson’s ratio (υ), Cauchy pressure (C″), shear constant (C′), anisotropic factor (A), Kleinman’s parameter (ζ), and Lame’s constants (λ and μ) are investigated using these elastic coefficients, as shown in Table 2. C_44_ > 0; C_11_ + 2C_12_ > 0; C_11_ − C_12_ > 0 [29] is a stability criterion for the cubic phase compounds. As a result of satisfying the above-mentioned physical criteria, the understudy compounds are mechanically stable.

From Table 2, the computed values of G_R_, Gv, and G_H_ for Ln_3_NIn (Ln = Dy-Lu) compounds lie in the range of 47.114 to 49.212, 61.498 to 70.884, and 55.236 to 58.999 GPa, respectively. From which it is obvious that Lu_3_NIn is more resistant to plastic deformation. B_o_ measures how much resistance a material provides in response to an external field [30,31]. The slight raise of pressure as volume drops is defined as B_o_. The computed value of B_0_ ranges from 119.765 to 135.121 GPa for all compounds. The results suggest that Lu_3_NIn is more resistive to external fields than the other compounds, which is also clear from Figure 5, regarding the cohesive energy and enthalpy of formation.

The ductile and brittle nature of the materials is explained by the Paugh ratio. When B_o_/G_H_ is larger than the critical value, the material is ductile, and when it is less than 1.75, it is brittle [32]. The Paugh ratios of these compounds are 2.163, 2.234, 2.299, 2.266, 2.286, and 2.290, which are greater than the boundary value, indicating that all of these compounds are ductile and none are brittle

The term “young modulus” (Y) is used to describe the stiffer nature of the material. The higher the value of Y, the stiffer the materials will be, and vice versa. The computed Y of all compounds spans from 157.531 to 181.002 GPa, indicating that they are stiffer, although Lu_3_NIn is more rigid than the others.

The Cauchy pressure is an additional parameter also used to explore materials’ ductile and brittle characteristics [33]. The +ive value of Cauchy pressure illustrates the ductile character of materials, and the −ive value shows their brittle nature. The estimated values for these compounds fall between 16.979 and 21.579 GPa, indicating that all compounds are ductile, as evidenced by the Paugh ratio.

The isotropic factor (A) of a material explains its isotropic behavior. This causes microscopic fissures in materials [34], with A = 1 for isotropic materials and A smaller or greater than 1 for anisotropic materials. The value of A for all compounds ranges from 2.664 to 3.841, indicating that they are all anisotropic in nature.

The Poisson’s ratio (υ) illustrates the compressibility of a compound. The υ, estimated from shear and bulk modulus for the present compounds, ranges from 0.276 to 0.286. The lower limit of the specified range points out that there is no alteration in the symmetry, while the upper value specifies whether a change in the volume of the material has occurred or not. Table 2 shows the computed values of Poisson’s ratios for the understudy compounds. It indicates that there is a variation in volume.

The Kleinman parameter (ζ) explains the material’s capacity to stretch and bond bending. Harrison [35] devised a formula based on elastic coefficients to calculate the Kleinman parameter. Kleinman showed that ζ = 0 represents bond bending and ζ = 1 represents bond stretching. The estimated Kleinman parameters for the entire compounds range from 1.030 to 1.163, suggesting that bond bending and stretching are feasible in all compounds, as specified by the ductile character of these compounds. As the value gets closer to 1, bond bending takes precedence over bond stretching.

Y and υ are used to calculate Lame’s constants (µ and λ). Lame’s constants vary mostly by the changing value of Y [36]. The low values of Y are responsible for the lower values of λ and µ. The determined Lame’s constants range from 61.498 to 70.884 and 78.767 to 87.864, signifying that all the compounds are rigid and stable. In summary, the mechanical stability, ductile, and anisotropic nature of compounds are confirmed by the elastic properties. The noteworthy attribute of metallic biomaterials, in contrast to other biomaterials like ceramics and polymers, is their ability to withstand tensile stresses, which may be exceedingly high and also dynamic in the case of metallic materials. This is the rationale behind the widespread use of metallic materials as structural materials for skeletal rebuilding when large applied loads are anticipated, such as those with adequate bending fatigue strength. Endo-prostheses for the hip and knee, plates, screws, nails, dental implants, etc., are typical examples of such heavily loaded implants [37,38]. These compounds are mechanically stable, load-bearing, and ductile, and therefore, can be the best materials to use as biomaterials.

### 3.4. Magnetic Properties

The magnetic characteristics of the materials are closely linked with the spin and orbital movement of the electrons. Exchange interactions of electrons are responsible for magnetism in magnetic compounds. Parallel alignment of individual magnetic moments creates ferromagnetism and anti-parallel alignment cancels their net effect, resulting in the anti-ferromagnetic nature of the materials [39,40].

**Figure 10:**
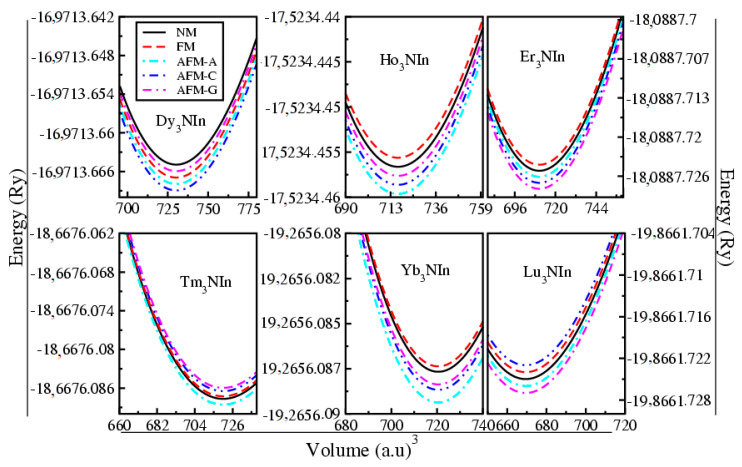
Stable magnetic phase optimizations of the rare earth nitrides Ln_3_NIn (Ln = Dy-Lu) anti-Perovskite.

In order to study the magnetic nature of Ln_3_NIn (Ln = Dy-Lu) anti-perovskites, ferromagnetic, non-magnetic, and A, C, and G type anti-ferromagnetic (AFM) magnetic arrangements are considered by taking double for each design, and their magnetic ground state energy against volume is shown in Figure 10.

Figure 10 shows that the ground state energy of the C-type AFM of Dy_3_NIn is smaller than the other magnetic phases, indicating that this compound shows the C-AFM stable magnetic phase. Moreover, the ground state energy of the A-type AFM of Ho_3_NIn, Tm_3_Nin, and Yb_3_NIn is smaller than the other magnetic phases, indicating that these compounds show the A-AFM stable magnetic phase and the ground state energy of G-type. The AFMs of Er_3_NIn and Lu_3_NIn aresmaller than the other magnetic phases, indicating that these compounds show the G-AFM stable magnetic phase.

The computed magnetic moments for Dy, Ho, Er, Tm, and Yb atoms in Ln_3_NIn (Ln = Dy-Yb) are presented in Table 3.

The magnetic moments of the Ln atom are 5.13, 3.66, 2.48, 1.413, and 0.973 µ_B_, respectively. The magnetic moment of the Ln atom decreases going from Dy to Yb due to the increasing number of electrons, while obeying Hund’s rules. Lu has a very low magnetic moment in the negative range and shows a paramagnetic nature, thus, it is non-magnetic in nature.

**Figure 11:**
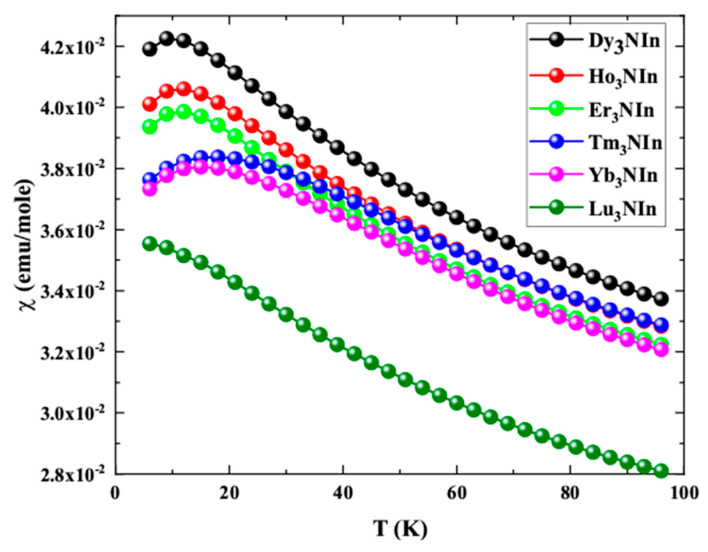
Magnetic susceptibility of the rare earth nitrides Ln_3_NIn (Ln = Dy-Lu) anti-Perovskite.

Boltzmann semi-classical transport theory [24] is used to determine the magnetic susceptibility (*χ*) for the understudy Ln_3_NIn (Ln = Nd-Tb) compounds embedded in the BoltzTraP program and is elucidated using the Curie–Weiss law [41] plotted in Figure 11.

**Figure 12:**
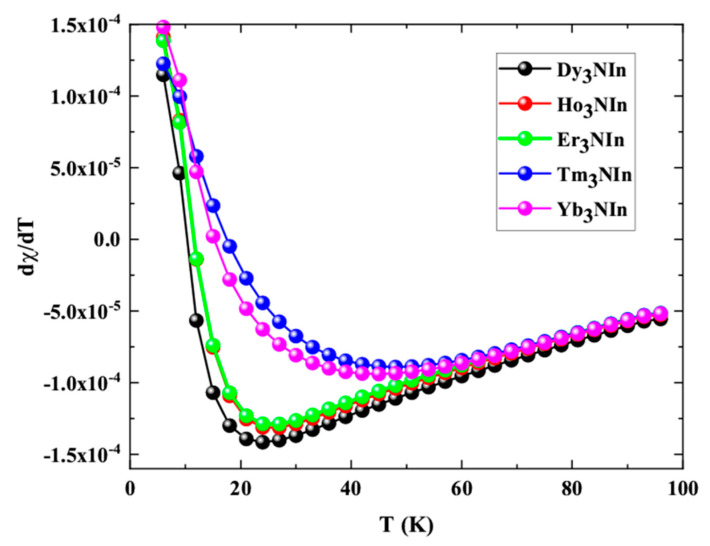
Derivative of magnetic susceptibility of the rare earth nitrides Ln_3_NIn (Ln = Dy-Lu)anti-Perovskite.

From the graph, it is clear that, at first, the susceptibility increases and then decreases, revealing the AFM nature of the understudy compounds. At 3 K, the calculated *χ* for Nd_3_NIn is 4.1910-2 emu/mole, for Pm_3_NIn is 4.0110-2 emu/mole, for Sm_3_NIn is 3.9310-2 emu/mole, for Eu_3_NIn is 3.7610-2 emu/mole, for Gd_3_NIn is 3.7310-2 emu/mole, and for Tb_3_NIn is 3.5510-2. The derivative of susceptibility with respect to temperature (d*χ*/dT) is taken and presented in Figure 12 to determine the Neel temperature (T_N_) for these compounds.

Figure 12 shows that the T_N_ values for Ln_3_NIn (Ln = Dy-Yb) compounds are 24, 27, 27, 48, and 45 K, respectively. In contrast, as the magnetic moments progress from Dy to Lu base compounds, the *χ* of the entire understudy compounds increases. Magnetic probes cannot read data accumulated in anti-ferromagnetic moments because of the anti-ferromagnetic feature of the underlying compounds, and data stays unaffected by disruptive magnetic fields and could be used as proper storage devices.

## 4. Conclusions

The structural, elastic, electrical, and magnetic properties of Lanthanide base Nitrides Ln_3_NIn (Ln = Dy-Lu) anti-perovskites have been investigated using DFT. The structure parameters agree with the experiment and disclose that the lattice parameters decrease going from Dy_3_NIn to Lu_3_NIn. The metallic character of all compounds is due to the f state of Ln with the addition of a minor effect of the d state elucidated from electronic properties and electrical resistivity. According to elastic characteristics, the understudy compounds are mechanically anisotropic, load-bearing, and ductile in nature.Because of their metallic and load-bearing nature, along with their ductility and bending fatigue strength, these materials will be used as structural materials for skeletal reconstructions. The magnetic moments of the Ln atom demonstrate that the Ln atom is effective in magnetism in these compounds. According to DFT and post-DFT calculations, all of the compounds are anti-ferromagnetic. This research opens a new route for the rational manufacturing of nitride base metallic AFM compounds by substituting Ln with other elements, which provides a fantastic area of research for varying properties.

## Figures and Tables

**Table 1:** The lattice constant, cohesive energy, enthlopy of formation and ground state energy of the anti-perovskite lanthanide nitrides Ln_3_NIn (Ln = Dy-Lu).

Compounds	GGA	GGA+U	HF	Exp [11]
**Dy_3_NIn**				
a_o_(Å)	4.744	4.775	4.856	4.785
E_o_(Ry)	−84,856.8	−84,856.7	−84,856.5	---
E_c_(Ry)	−32.4056	−32.2442	−32.1113	
ΔH(Ry)	−6.48111	−6.44884	−6.42226	
**Ho_3_NIn**				
a_o_(Å)	4.728	4.747	4.804	4.761
E_o_(Ry)	−87,617.5	−87,617.2	−87,617.1	---
E_c_(Ry)	−33.1698	−32.8644	−32.7608	
ΔH(Ry)	−6.63396	−6.57288	−6.55216	
**Er_3_NIn**				
a_o_(Å)	4.684	4.729	4.769	4.736
E_o_(Ry)	−90,444.2	−90,443.9	−90,443.2	---
E_c_(Ry)	−33.6942	−33.3484	−32.6942	
ΔH(Ry)	−6.73884	−6.66968	−6.53884	
**Tm_3_NIn**				
a_o_(Å)	4.644	4.7074	4.731	4.708
E_o_(Ry)	−93,338	−93,337.7	−93,337.6	---
E_c_(Ry)	−34.1974	−33.8687	−33.7474	
ΔH(Ry)	−6.83949	−6.77375	−6.74947	
**Yb_3_NIn**				
a_o_(Å)	4.632	4.684	4.718	---
E_o_(Ry)	−96,301.1	−96,301	−96,299.9	---
E_c_(Ry)	−35.7531	−35.7116	−34.6074	
ΔH(Ry)	−7.15062	−7.14231	−6.92148	
**Lu_3_NIn**				
a_o_(Å)	4.621	4.663	4.695	4.671
E_o_(Ry)	−99,330.9	−99,330.9	−99,330.8	---
E_c_(Ry)	−36.1007	−36.0341	−35.9532	
ΔH(Ry)	−7.22014	−7.20682	−7.19063	

**Table 2:** Estimated elastic moduli C_ij_, Voigt’s (G_v_) shear modulus, Reuss’s(G_R_) shear modulus, Hill’s shear modulus (G_H_), Bulk modulus (B_O_),Pugh-ratio(B_O_/G),Young modulus (Y), shear constant (C′), Cauchy-Pressure (C″),Anisotropy constant (A), Poisson’s ratio (υ), Kleinman parameter (ζ) and Lame’s coefficients (λ and μ) of the anti-perovskite lanthanide nitrides Ln_3_NIn (Ln = Dy-Lu).

Parameters	Dy_3_NIn	Ho_3_NIn	Er_3_NIn	Tm_3_NIn	Yb_3_NIn	Lu_3_NIn
C_11_	160.785	162.89	165.874	167.689	168.365	170.059
C_12_	99.256	103.671	109.562	111.026	114.76	117.651
C_44_	81.987	83.761	87.983	91.528	96.629	100.672
G_V_ (GPa)	61.498	62.1	64.052	66.249	68.698	70.884
G_R_ (GPa)	49.212	48.373	47.559	48.369	47.318	47.114
G_H_ (GPa)	55.355	55.236	55.806	57.309	58.008	58.999
B_O_ (GPa)	119.765	123.411	128.333	129.914	132.629	135.121
B_O_/G	2.163	2.234	2.299	2.266	2.286	2.29
Y (GPa)	157.531	159.54	164.748	169.873	175.75	181.002
C′	30.764	29.609	28.155	28.331	26.802	26.203
C″	17.268	19.91	21.579	19.498	18.131	16.979
A	2.664	2.828	3.124	3.23	3.605	3.841
ν	0.28077	0.284	0.286	0.282	0.279	0.276
ζ	1.03	1.063	1.106	1.109	1.144	1.163
µ	61.498	62.1	64.052	66.249	68.698	70.884
λ	78.767	82.011	85.631	85.747	86.83	87.864

**Table 3:** Calculated values of magnetic moments per Ln atom in units of (µ_B_) of the anti-perovskite lanthanide nitrides Ln_3_NIn (Ln = Dy-Lu).

Parameters	Dy_3_NIn	Ho_3_NIn	Er_3_NIn	Tm_3_NIn	Yb_3_NIn
µ_eff_/Ln T_N_ (K)	5.13 24	3.66 27	2.48 27	1.413 48	0.973 45

## Data Availability

Not applicable.
